# Early Introduction of Power Mobility Devices for Children with Fukuyama Congenital Muscular Dystrophy and Its Psychological Impact on Caregivers: A Case Report

**DOI:** 10.3390/pediatric15030037

**Published:** 2023-07-05

**Authors:** Hitomi Fujita

**Affiliations:** Department of Rehabilitation, Faculty of Health Sciences, Nihon Fukushi University, 26-2 Higashihaemi-cho, Handa 475-0012, Aichi, Japan; hitomifu@n-fukushi.ac.jp; Tel.: +81-569-20-0131 (ext. 9116)

**Keywords:** power mobility device, Fukuyama congenital muscular dystrophy, psychological effect, assessment of learning powered mobility use tool, early intervention, physical therapy

## Abstract

Recently, motorized mobility devices (or power mobility devices (PMDs)) have been introduced for infants and toddlers who lack the means for self-mobility. Previous reports have primarily focused on PMDs for individuals with cerebral palsy. Few have explored PMDs for individuals with neuromuscular diseases who have intellectual disabilities. This report presents a case study of the early introduction of a PMD for an infant with Fukuyama congenital muscular dystrophy and presents the results of an interview with the father regarding psychological aspects and the child’s manipulative abilities. The PMD was introduced at the age of 1 year and 10 months, and the changes during the 19 months after the introduction were evaluated six times, using the Assessment of Learning Powered mobility use tool (ALP). A semi-structured interview with the father was conducted 19 months after the introduction. The ALP evaluation and the interview were conducted by one physical therapist and two physical therapy students, and the results were shared with the hospital’s physical therapist and nurses at the nursing facility. This report provides a basis for expanding the scope of PMD use and for considering the family’s involvement, especially for the child.

## 1. Introduction

From the perspective of the International Classification of Functioning (ICF), Disability and Health, and the “F-words” of childhood disability, approaches to children and their families have expanded to encompass various efforts [1]. A comprehensive developmental support program can help enrich the lives of developing children with disabilities and enable them to actively expand their own world of activity.

An important chance for children’s self-directed physical activity and social interaction is the introduction of power mobility devices (PMDs). A series of case studies have been conducted involving children with severe motor disabilities, and several systematic reviews have examined the effectiveness and measurement methods of PMDs. In a prior systematic review [2], abundant qualitative changes were reported, including successful participation in activities of daily living and the enjoyment of PMD use. They also described environmental and technological factors and longer training strategies as worthy of further experimental study. Field and Livingstone (2018) reviewed the measures and clinical applications of PMD skill acquisition and reported convergence with the Powered Wheelchair Mobility Program (PMP) and the Assessment of Learning Powered mobility use (ALP) as the primary measures [3]. Although both these measures are in the early stages of development, and thus have limited use, the PMP is a task-based measure [4], and the ALP is a process-based measure [5]. Gefen et al. [6] demonstrated good reliability and validity for the PMP and ALP.

Concerning PMDs, a systematic review—which examined the effectiveness of training methods—measured outcomes related to goals and indicated that a combined approach incorporating play and the natural environment was associated with the strongest positive results [7]. Most participants in the 27 studies extracted in their review examined children with motor disabilities such as cerebral palsy, spinal muscular atrophy, congenital polyarthritis, or Down’s syndrome. A systematic review of 23 studies on modified ride-on cars—commercially available toy cars fitted with switches and seats at a lower cost than traditional electric mobility devices—found from the ICF framework that activity and participation, as well as families’ understanding of their children’s abilities, has positive impacts [8]. However, most of the results from each report were descriptive, lacking quantitative synthesis and summary measures.

Consequently, the Rehabilitation Engineering and Assistive Technology Society of North America recommended early use of PMD for children with mobility limitations [9]. In 2020, the EMPoWER project of the United Kingdom’s National Institute for Health Research strongly supported the positive impact of PMD interventions on children’s movement and activity [10]. There was also moderate support for children’s participation, play, social interaction, and the impact of accidents and pain on safety.

Interventions using PMD are likely to have a positive impact on children’s development. However, the ethical difficulties of setting up a target group and the impracticality of homogenizing groups have been noted as complicating the application of randomized controlled trials (RCTs) that consider the establishment of evidence [11].

There may, however, be a way to clarify the efficacy indices of PMDs by accumulating practical reports and utilizing clinical evidence. The demand for PMDs is increasing, not only for children but also for adults, because of the increase in the number of people with mobility difficulties and the ICF’s viewpoint. PMD use promotes autonomy, independent living, and social participation in all aspects of life [12], and PMD driving requires complex human (social and cognitive factors), environment, and device-related interactions. Because of said interactions, the considerations of healthcare professionals are vital. Cognitive function is the most challenging issue. A systematic review concerning the impact of cognitive function on PMD use revealed that cognitive function is necessary for PMD use [13]. Furthermore, based on the effect of early provision of electric wheelchairs to children with tetraplegia [14], the achievement of independent use of PMD is related to intelligence quotient (IQ), and the relationship between a child’s cognitive function is considered. Regarding the investigation of the role of cognition in PMD use in adults, future strategic research priorities were identified at a consensus workshop [15]. While research on the introduction of PMD is progressing among adults, the cognitive function of adults and the IQ and intellectual ability of children should not be treated the same way, and interpretation that fully considers each characteristic is required.

### Objective

The first objective was to clarify the process of qualitative change in mastery through a case study of a child with both intellectual and physical disabilities. The author aimed to clarify that an intervention focusing on the perception of physicality, such as body perception, body ownership, and motor subjectivity, while freely moving may promote an understanding of the interaction between oneself and the environment. Second, based on the interview, the author sought to understand the psychological aspects of how the father’s perspectives shifted when PMDs were introduced.

## 2. Methods

### 2.1. Participants

This report is a case study. The participants were a girl aged 1 year and 10 months (at the start of PMD introduction) diagnosed with Fukuyama congenital muscular dystrophy, and her father. Her parents hoped to have access to an electric wheelchair for her in the future.

At three months of age, she was diagnosed with developmental delay and generalized hypotonia at a specialized institution. She started outpatient physical and occupational therapy (twice a month) at six months of age. She was using a personalized chair to sit and a backrest that promoted head control. No further motor function was observed. The patient was either carried in her parents’ arms or pushed in a stroller for transportation. Communication was mainly babbling and pointing, and she spoke fewer than 10 words. Since her mother worked during the day, her father was mainly responsible for her daily care and hospital visits.

### 2.2. Data Collection

#### 2.2.1. Video Collection

Video footage taken once every three months was used to assess PMD. When she was using PMD at home, videos were shot by her parents and shared with the author. At the rehabilitation facility, the author shot the videos. Both shots were taken for about 10 min just before she got on the PMD and taken once every 3 months.

#### 2.2.2. Semi-Structured Interview Guide

A semi-structured interview was conducted using the following four items as a guide.

(1) How you felt when you decided to introduce PMDfor the first time?
(2) Please tell us what you think about PMD maintenance.
(3) Do you have any feelings about your child’s driving skills compared to before the introduction?
(4) Please tell us about the stress you as parent feel in your life.

### 2.3. Intervention Details

#### Introduction of PMD (Figure 1)

Babyloco, a PMD available in Japan that was developed based on a proposal by the Kids Loco Project [16]—a private organization promoting electric mobility devices in Japan (Mobility Aids for Children)—was used. This study required no copayment from the participant’s family. A joystick was used to control the PMD, and it was equipped with a sitting chair, which is a standard product.

**Figure 1:**
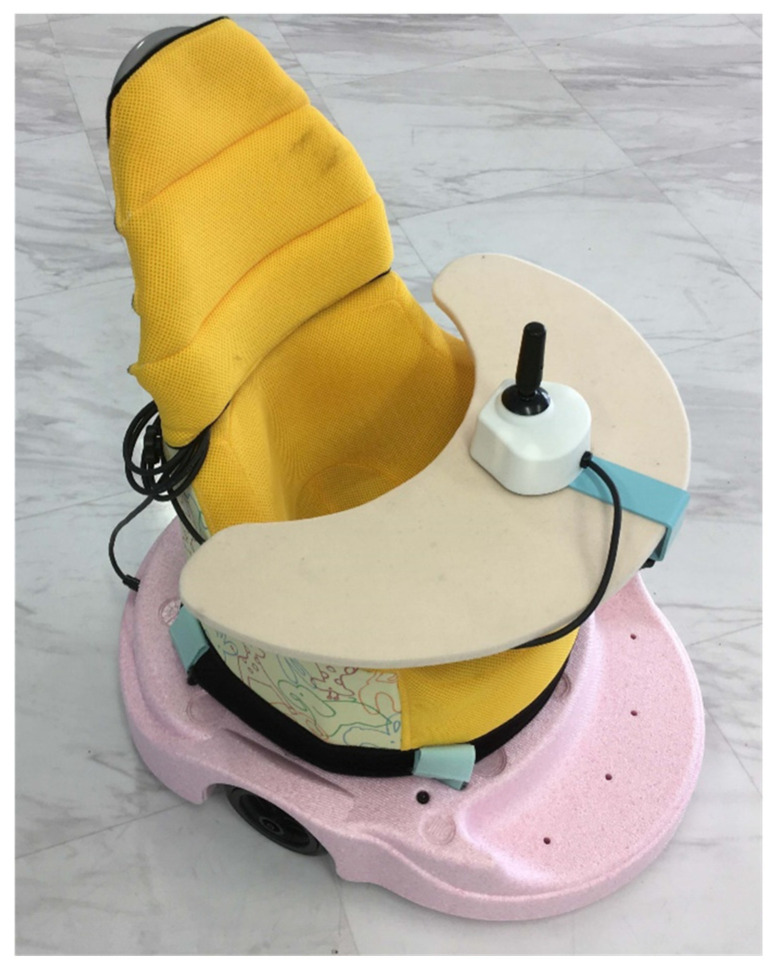
Power mobility device used in this case “Babyloco”.

### 2.4. Procedure (Table 1)

The patient’s parents obtained information about PMDs from a patient group. When the patient was 1 year and 10 months old, her parents were referred by their physiotherapist to this study’s author for an introduction to PMD. This was the patient and the author’s first meeting.

For the first two months, practice was conducted during outpatient physical therapy. After adjustments were made to equipment settings, sitting posture, table position, and other factors, the equipment was loaned for use in the patient’s home. Thereafter, periodic checkups were conducted monthly to evaluate the operation status and check for any problems related to equipment use.

The child’s operating ability was assessed using the Japanese version of ALP–instrument [17], and explanations and guidance were provided to the parents based on the Japanese version of ALP Facilitating Strategies, version 2.0 [17], according to the ALP level.

**Table 1:** Time–series changes due to PMD intervention.

Intervention Period (Months)	Age (Y/M)	Place of Use	Adjustment of PMD	Frequency of Use	ALP–Tool *
1	1Y10M	Outpatient	Initial setting table height chair position	1/w, 20 min	1–2
2	1Y11M		Joystick change (from standard type to small size) Added backrest extension parts for sitting chair		
3	2Y	Home	Repositioning the controller Attaching the sole installation base Raising the seat	1/w, 20 min	3–5
4	2Y1M				
5	2Y2M		As a countermeasure for knee extension, fix the lower leg and chair with an elastic wrap.	15 min/w	
6	2Y3M				
7	2Y4M				
8	2Y5M				
9	2Y6M			rarely	3–5
10	2Y7M	Nursing school	Table size change (extended type → standard size)	2/w, 10 min	
11	2Y8M		Explanation to nursing staff Halve the height of the sole installation base Release the raising of the seat		5–6
12	2Y9M	Hospitalized for 3 weeks		none	
13	2Y10M		Added footrest extension parts	2/w, 10 min	
14	2Y11M				
15	3Y				7–8
16	3Y1M		Battery exchange		
17	3Y2M				
18	3Y3M				
19	3Y4M		Increase speed and acceleration to medium level Extending the backrest of a sitting chair	3/w, 15–30 min	8
20	3Y5M				

* ALP–tool: ALP instrument and facilitating strategies.

### 2.5. Analysis

#### 2.5.1. Assessment and Intervention Methods (from Video Footage of the ALP)

All video images and statements were verbally coded and qualitatively analyzed using Steps for Coding and Theorization Analysis [18] (SCAT); furthermore, data were analyzed separately by the author and two physical therapy students. SCAT is a 4-step coding process used to identify themes and constructs, and to develop a storyline by weaving these themes and constructs together. The patient’s ALP level was determined by comparing the results of the SCAT with the ALP. The ALP level was based on the theoretical description derived from the SCAT analysis. It was defined as the closest match between the content of the theoretical description and the content described in the ALP. This level was determined by the author, who analyzed the SCAT, and by two physical therapy students. When the level was difficult to determine, the same three people as above were consulted, based on the theoretical description and videos, until a consensus was reached. In addition to the ALP, the patient’s parents were advised according to the ALP Facilitating Strategies (version 2.0) to promote awareness of her use of tools, and the sitting environment was adjusted by the author.

This case study focused on the use of PMD in daily life as a realistic approach rather than a special program for future PMD development. Therefore, the duration and frequency of use were not specified in advance, and information on actual use was collected during the periodic checkups.

#### 2.5.2. Adoption of the ALP

In 2011, Nilsson et al. developed the ALP as a therapeutic intervention that provides children and adults who have severe cognitive impairment with the opportunity to learn new skills and is recommended for use with training strategies [19]. It is currently considered the only measure that covers all stages of the learning process.

The prime reason for choosing the ALP [5] for this case study was that the patient was introduced to powered mobility equipment very early in life. Therefore, it was felt that an assessment and treatment strategy that focused on the early stages of learning, such as slight changes in joystick operation, was necessary. The ALP has excellent convergent validity in the PMP and the Powered Mobility Proficiency Test [6]. Because of her intellectual disability, it was decided to employ a Japanese version of the ALP process-based assessment rather than a task-based assessment such as the PMP.

#### 2.5.3. Interview with the Father

Eighteen months after the introduction of the PMD, a semi-structured interview was conducted with the patient’s father, who was her primary caregiver and most familiar with her life. The interview guide consisted of questions on (1) what the father expected from the introduction of the PMD, (2) changes in communication within the family, (3) the child’s driving skills, and (4) changes in the parents’ lives. An interview guide was developed by the author, and the interview was conducted by two physical therapy students. The interview lasted approximately 30 min, with different sections based on the environments in which the PMD was used. The interview was recorded using a voice recorder, transcribed verbatim, and then qualitatively analyzed using SCAT, by the author and two physical therapy students, in the same way as the assessment of the videos of the patient.

The interview was conducted with the approval of the Ethical Review Committee on “Research Involving Human Subjects” of the authors’ affiliate institution. The patient’s PMD change was recorded on video and analyzed as part of this study. Prior to conducting the interview with her father, a written explanation was shared with the parents, after which informed consent was obtained.

## 3. Results (Table 2)

As the first objective of this study, Table 2 shows the changes in the ALP phase across the intervention period (months). Data were collected from six videos. When the patients’ understanding of the ALP, attention, activities, movement, and tool use reached Level 5, communication had not progressed from Level 3. The environment for implementing the ALP facilitation strategies was examined, and the policy was converted to use in the daycare facility. One month after the start of use in the daycare facility, the overall ALP level increased to Levels 5–6. Furthermore, one year and two months after introduction, when the child was three years old, her ALP was at Levels 7–8. Tool comprehension and activity reached Level 8 in approximately 15 months, and cognitive function and communication reached Level 8 in approximately 19 months. It was confirmed that “expressions and emotions” temporarily decreased nine months after PMD introduction. As the patient became more proficient, the levels tended to converge to a single phase.

**Table 2:** Change in ALP level after intervention of PMD.

Month(s)	Attention	Activity & Movement	Understanding of Tool Use	Expressions & Emotions	Interaction & Communication
1	2	2	1	1	2
3	5	6	3	4	3
9	5	5	5	3	3
11	5	6	6	6	5
15	8	8	8	7	7
19	8	8	8	8	8

### 3.1. Adverse or Unanticipated Events That Interfered with the Intervention

At 2 years and 9 months, the patient required hospitalization and treatment for three weeks owing to a respiratory syncytial virus infection. During hospitalization, she did not use the PMD and spent most of her time lying on her bed or riding in a stroller. At the time of her hospitalization, her physical function temporarily deteriorated, and she had difficulty maintaining a sitting position. However, by the time she was discharged from the hospital, she had recovered to her pre-hospital physical functions. Because the patient was unable to use the PMD for a certain period during the hospitalization, a battery problem occurred, due to disuse. The supplier resolved this problem by replacing the battery.

### 3.2. Interview with the Father (Table 3 and Table S1)

Table 3 shows the content analysis of the interview as a result for the second purpose. The father’s own psychological changes were divided into five periods, and the following constructs were generated (Theoretical description): (1) before the introduction of the PMD, the father expressed “anxiety about the child’s future;” (2) immediately after the introduction of the PMD, he expressed “expectations for the child to gain experience in moving by herself;” (3) during PMD practice, he was “surprised by the child’s rapid growth in operating skills” and was “aware of the need for teamwork;” (4) approximately 10 months after the introduction of the PMD, “doubts about the child’s proficiency in operating the equipment” were shown; and (5) after the transition to a daycare facility, his wishes related to his child’s operation becoming goal-oriented driving unexpectedly came true, and “a change in concept from relative to absolute evaluation of the child” was confirmed.

**Table 3:** Analysis results using an interview guide and SCAT in semi–structured interviews (partial excerpt).

	Text	<4>Themes and Compositional Concepts
(1) First time the PMD	My child had a muscular disease called Fukuyama muscular dystrophy, which was diagnosed when she was three months old, and we found out that something was wrong when she went to pediatric hospital. We were groping around, and it was the first time for us, so we didn’t know what to expect. We were groping our way through it all, and it was the first time for us, so we didn’t know what to expect, but as we were promoting her growth, we found that we were connected with people all over the country who had the same symptoms, so we happened to find out about Baby Loco at the same time. We happened to know about Baby Loco at the time when we wanted to buy it, but as Ms. Fujita said before, we had to purchase it at our own expense, so it was difficult for us to get hold of it. I happened to mention it to the physical therapist at pediatric hospital, and she told me that there was someone who wanted to use it, so it was really perfect timing for us. So we thought, well, this really fits the bill, so we’d really like to borrow it, so we’re going to use it. So, we were like, well, if we can provide that kind of information, if it’s good enough for our child, we’d be happy to use it, so we were like, we’d love to use it at the right time. Yes.	Explanation of how the disability was discovered/ Diagnosis of intractable disease and budding anxiety about the future/ Emergence of the barrier of information refugees/ Encounter with unapproved devices guided by empirical knowledge/ Willingness to work together in an interactive manner
(2) PMD maintenance	I had the battery changed once, but I didn’t think that the battery would be damaged so much if I didn’t have knowledge about it, and also, at the beginning, I had to add more footholds and lift her legs. I had to add some bulk to the foo–holds and other places in the beginning, and I had to raise her legs and so on, so it was very difficult to adjust those places at first. Now that they have done a lot of work, I have nothing more to say about it, but rather than maintenance, I guess, at the beginning, I was trying to find out how to make her ride in the right position and how to make her legs float to make it easier for her to ride this time.	Accumulation of collaborative work with experts/ Difficulty in adjusting equipment through trial and error/ Awareness of need for expertise/ Sustainability of collaborative work
(3) Driving skills	Ah, well, that’s just the way it is. It’s obvious, isn’t it? I’m still in the middle of being surprised. I don’t think I even knew what that lever was doing at the beginning, and I probably wasn’t even interested in it in the first place, or even tried to touch it. I just sat there and felt like there was something there. I didn’t even think I could operate it by herself. So before the nursing school, she was still practicing operation with the PT at the hospital. And she was able to move forward and backward little by little, but as you can see, she didn’t want to go there in the first place. She don’t think she were really happy to be ridden, or she didn’t remember the fun of it, or she wondered why she was being ridden and going around in circles like this. So, yes, until this point, she really only had the feeling that she wanted to go forward on our own volition. I think it was after the introduction of the nursing school, which, to be honest, I had not yet ridden that many times at home. We were really surprised, too. We didn’t think she would be able to operate it to such an extent. We happened to bring it home to recharge it, and when we let her ride it at home and saw this scene, we were really surprised. But this is my younger daughter, and she moved to my younger daughter’s place by herself, of her own volition. I was also able to see how she could operate it, for example, by moving forward and sideways while changing direction, but compared to the first time, I could see that she really wanted to go where she wanted to go on her own. So, I think that now, she is touching it as if it was one of her own means of action.	<Before nursing school, before introduction> Guidance to doubts about proficiency in equipment operation during the stagnant learning period. <After introducing it to nursing school> Surprise at the rapid growth of operating technology /Automation of active movement
(4) Stress in parents’ lives	Well, stress, well, there is not much I can do about it, well, I don’t really think about it like this, but I am really glad that we introduced it, because now she can play with my younger daughter more, and by being able to do it like this....... If she had been sitting like this, she wouldn’t have been able to approach them and play with them. So, in that sense, I felt that we were able to create an environment where the sisters could get along well with each other. I don’t feel too stressed. On the contrary, I now think that it was a good thing as I said before.	Emergence of positive changes in relationships, etc./ emergence of active involvement in the surrounding environment
**Theoretical description**		
Parents of children with congenital disabilities may expect that the introduction of equipment will provide practice for future mobility and that the child will gain mobility experience on her own. Immediately after the introduction of the equipment, we sometimes feel that the children do not understand the situation when they ride on the unknown equipment, and we sometimes feel that there is no change in the children’s driving skills compared to before and immediately after the introduction of the equipment. After the introduction of the equipment, the operability of the equipment improves and the child begins to understand the mechanism of the equipment after the equipment is adjusted. The environment changes, and the child’s driving skill improves significantly, and the child’s needs for daily mobility behavior may be felt. Comparing the initial introduction with the present, the father may feel a significant growth in the child’s driving skills, such as the emergence of goal-oriented driving, and these successful experiences may lead to unexpected conceptual changes and joyful.		

## 4. Discussion

The present results support including PMDs in the intervention of Fukuyama congenital muscular dystrophy—a neuromuscular disease and intellectual disability—as well as in cerebral palsy [6,20,21,22,23,24,25,26], spina bifida [27], and spinal muscular atrophy [28]. Based on the experience of skill acquisition in this case, it is necessary to expand the scope of PMD use and consider the period required to reach proficiency.

The findings of this study also revealed that ALP evaluation can be used in parallel with prompting strategies. In the process of recording ALP evaluation over time, multiple phases of behavior were observed immediately after the introduction of the PMD, which gradually converged as the patient became more proficient. The switch from home use to daycare facility use while monitoring changes in ALP levels may have contributed to the improvement in ALP levels. In this study, the fact that the changes observed over a four-month period at home were observed within one month at the daycare facility may be due to various factors, including the influence of the difficulty level of ALP, frequency of use, timing of introduction, and the environment. As for the temporary decline in “expressions and emotions”, there may be a disconnect between locomotion and socialization until the child sees the locomotion device as a means to a social end, as reported in a study of children with cerebral palsy [25].

In the temporal changes of the ALP, comparative information—such as time factors and the order of movement among the five levels—is required. In addition, it may be necessary to understand the characteristics of the treatment strategy for each case, especially in the early stages of the introduction, so that the evaluator is not confused. Simultaneously, as mentioned in Field and Livingstone [3], the current results should not be used in pre-introduction decisions.

Regarding the growth response of the equipment and physical function of the child, it was essential to create an environment in which the child could easily demonstrate her abilities and adjust the equipment according to her motor functions (selection and fine-tuning of the equipment, such as seat holding, joysticks, and tables). The timely involvement of specialists, such as physical therapists, is important [29].

Regarding the father’s psychological change, it was confirmed that his anxiety about the child’s disability, which was expressed immediately before the introduction of the PMD, changed to an expectation of the child’s acquisition of mobility. After PMD introduction, the father’s doubts were confirmed again. Then, as his child’s driving skills improved when the PMD was switched to a daycare facility, the father again showed positive feelings. This has previously been seen among parents with children with disabilities [30], and both positive and negative emotions are expected to surface, time and again.

As a characteristic of the neural network during development, the number of neurons decreases, and the number of synapses increases, with myelination of the central nervous system (motor cortex) during the acquisition process of gross motor development. The number of synapses required for the cranial nerve network reaches its highest point at 3–4 years of age. During this period, experiences and environment greatly influence later development, enabling children to interact with people, objects, and the environment in various ways [31]. In the current case study, growth in emotion and communication while acquiring new mobility skills was also confirmed between the ages of two and three years [32]. Because external information and motor sensation are integrated during the developmental process, this patient, who had no motor experience, may have acquired the process of converting perceptual information into motor information.

In this study, a case of PMD implementation in an infant with intellectual and physical disabilities, for which information was lacking in previous reports, is reported. The author discusses this case as a feasibility study to clarify clinical questions for future RCTs. Additionally, the results provide useful information on the expansion of the number of PMD participants; the qualitative changes in activities, including the time factor until acquisition; and the psychological changes necessary for the relationship with caregivers.

### Methodological Considerations

A limitation of this study is that it is a case report; thus, these preliminary findings should be replicated/confirmed by future large-scale, better-controlled studies. Employing a control group may be useful in testing whether changes are owing to natural progression and development or the specific child recruited. The author also aimed to confirm whether ALP captures these improvements accurately and whether there are correlations between ALP and other developmental measures that assess young children’s physical, cognitive, and social development. To this end, the inclusion of different scales (assessing skill progress and other physical and developmental changes) can add a more multifaceted perspective. Further, the introduction of PMDs in the daycare setting was not set up to the point where they were shared in the context of interacting with younger children for accident-prevention purposes. It was necessary to limit the use of PMDs to a specific space—where safety was confirmed and where the class teacher was present. Although the environment of use should be expanded to permeate children’s lives, it was difficult to do so in this case because of the risk management of the caregivers and the environment. As inclusive education becomes more widespread [33], it is necessary to consider the above issues and the spread of PMD use.

However, it is noteworthy that this case showed changes, even in a limited setting of use. This report provides sufficient information to encourage the introduction of PMDs for children and their families considering their use in the future.

## 5. Conclusions

Early introduction of PMD brought about effective changes in an infant with Fukuyama congenital muscular dystrophy. It was also shown that there was a beneficial change in the psychological aspect of the caregiver. Professionals need to build relationships with parents in family-centered care [34] while recognizing that these psychological conditions are part of the process of various adaptations. It was difficult to explain the prospects of learning about PMDs to the parents in this case because obtaining information on any previous case was difficult. The author believes this report provides useful information for similar cases in the future.

## Data Availability

The data that support the findings of this study are available from the corresponding author, Hitomi Fujita, upon reasonable request.

## Supplementary Materials

The following supporting information can be downloaded at: https://www.mdpi.com/article/10.3390/pediatric15030037/s1, Table S1: Analysis results using an interview guide and SCAT in semi-structured interview.

## References

[1] Rosenbaum P., Gorter J.W. (2012). The ‘F-words’ in childhood disability: I swear this is how we should think!. Child Care Health Dev..

[2] Livingstone R., Field D. (2014). Systematic review of power mobility outcomes for infants, children and adolescents with mobility limitations. Clin. Rehabil..

[3] Field D.A., Livingstone R.W. (2018). Power mobility skill progression for children and adolescents: A systematic review of measures and their clinical application. Dev. Med. Child Neurol..

[4] Furumasu J., Guerette P., Tefft D. (1996). The development of a powered wheelchair mobility program for young children. Technol. Disabil..

[5] Nilsson L., Durkin J. (2014). Assessment of learning powered mobility use—Applying grounded theory to occupational performance. J. Rehabil. Res. Dev..

[6] Gefen N., Rigbi A., Weiss P.L.T. (2022). Reliability and validity of pediatric powered mobility outcome measures. Disabil. Rehabil. Assist. Technol..

[7] Kenyon L.K., Hostnik L., McElroy R., Peterson C., Farris J.P. (2018). Power mobility training methods for children: A systematic review. Pediatr. Phys. Ther..

[8] Hospodar C.M., Feldner H.A., Logan S.W. (2021). Active mobility, active participation: A systematic review of modified ride-on car use by children with disabilities. Disabil. Rehabil. Assist. Technol..

[9] Rosen L., Plummer T., Sabet A., Lange M.L., Livingstone R. (2023). RESNA position on the application of power mobility devices for pediatric users. Assist. Technol..

[10] Bray N., Kolehmainen N., McAnuff J., Tanner L., Tuersley L., Beyer F., Grayston A., Wilson D., Edwards R.T., Noyes J. (2020). Powered mobility interventions for very young children with mobility limitations to aid participation and positive development: The EMPoWER evidence synthesis. Health Technol. Assess..

[11] Salminen A.L., Brandt A., Samuelsson K., Töytäri O., Malmivaara A. (2009). Mobility devices to promote activity and participation: A systematic review. J. Rehabil. Med..

[12] Brandt A., Iwarsson S., Ståhle A. (2004). Older people’s use of powered wheelchairs for activity and participation. J. Rehabil. Med..

[13] Pellichero A., Kenyon L.K., Best K.L., Sorita É., Lamontagne M.E., Lavoie M.D., Routhier F. (2020). Influence of cognitive functioning on powered mobility device use: Protocol for a systematic review. JMIR Res. Protoc..

[14] Bottos M., Bolcati C., Sciuto L., Ruggeri C., Feliciangeli A. (2001). Powered wheelchairs and independence in young children with tetraplegia. Dev. Med. Child Neurol..

[15] Best K., Smith E., Pellichero A., Sorita E., Archambault P., Kenyon L., Lamontagne M.E., Lemelin B., Kirby R.L., Routhier F. (2023). International research priorities on the role of cognition in power mobility device use: In pursuit of informed clinical practices and knowledge translation. Assist. Technol..

[16] Kids Loco Project. http://www.mech.usp.ac.jp/~maw/KLP2016/home.html.

[17] Assessment of Learning Powered Mobility Use-Facilitating Strategies 2014. https://www.lisbethnilsson.se/en/alp-tool/alp-tool-in-japanese/.

[18] Otani T. (2008). “SCAT” a Qualitative Data Analysis Method by Four-step Coding: Easy Startable and Small Scale Data-applicable Process of Theorization. Bull. Grad. Sch. Educ. Nagoya Univ..

[19] Nilsson L., Eklund M., Nyberg P., Thulesius H. (2011). Driving to learn in a powered wheelchair: The process of learning joystick use in people with profound cognitive disabilities. Am. J. Occup. Ther..

[20] Feldner H. (2019). Impacts of early powered mobility provision on disability identity: A case study. Rehabil. Psychol..

[21] Furumasu J., Guerette P., Tefft D. (2004). Relevance of the Pediatric Powered Wheelchair Screening Test for children with cerebral palsy. Dev. Med. Child Neurol..

[22] Jones M.A., McEwen I.R., Neas B.R. (2012). Effects of power wheelchairs on the development and function of young children with severe motor impairments. Pediatr. Phys. Ther..

[23] Livingstone R., Field D., Sanderson C., Pineau N., Zwicker J.G. (2022). Beginning power mobility: Parent and therapist perspectives. Disabil. Rehabil..

[24] Nisbet P.D.J.T. (2002). Assessment and training of children for powered mobility in the UK. Technol. Disabil..

[25] Ragonesi C.B., Chen X., Agrawal S., Galloway J.C. (2011). Power mobility and socialization in preschool: Follow-up case study of a child with cerebral palsy. Pediatr. Phys. Ther..

[26] Sonday A., Gretschel P. (2016). Empowered to play: A case study describing the impact of powered mobility on the exploratory play of disabled children. Occup. Ther. Int..

[27] Lynch A., Ryu J.C., Agrawal S., Galloway J.C. (2009). Power mobility training for a 7-month-old infant with spina bifida. Pediatr. Phys. Ther..

[28] Dunaway S., Montes J., O’Hagen J., Sproule D.M., Vivo D.C., Kaufmann P. (2013). Independent mobility after early introduction of a power wheelchair in spinal muscular atrophy. J. Child Neurol..

[29] Kenyon L.K., Jones M., Breaux B., Tsotsoros J., Gardner T., Livingstone R. (2020). American and Canadian therapists’ perspectives of age and cognitive skills for paediatric power mobility: A qualitative study. Disabil. Rehabil. Assist. Technol..

[30] Nakata Y.J.W.P.R. (1995). A parental response to having a child with developmental disorders: A stage model or chronic sorrow?. Waseda Psychol Rep..

[31] Hadders-Algra M. (2018). Early human motor development: From variation to the ability to vary and adapt. Neurosci. Biobehav. Rev..

[32] Huang H.H., Ragonesi C.B., Stoner T., Peffley T., Galloway J.C. (2014). Modified toy cars for mobility and socialization: Case report of a child with cerebral palsy. Pediatr Phys Ther..

[33] Szumski G., Smogorzewska J., Grygiel P. (2022). Academic achievement of students without special educational needs and disabilities in inclusive education-Does the type of inclusion matter?. PLoS ONE.

[34] King G., Williams L., Hahn Goldberg S. (2017). Family-oriented services in pediatric rehabilitation: A scoping review and framework to promote parent and family wellness. Child Care Health Dev..

